# Effectiveness of Plyometrics Along With Pilates Exercises in Increasing Vertical Jump Performance Among Basketball Players

**DOI:** 10.7759/cureus.32957

**Published:** 2022-12-26

**Authors:** Rahul Chouhan, Anand Misra, Rajat Soni, Ashish Joseph, Roshan Umate

**Affiliations:** 1 Physiotherapy, Sri Aurobindo Institute of Allied Health and Paramedical Sciences, Sri Aurobindo University, Indore, IND; 2 Research and Development, Jawaharlal Nehru Medical College, Datta Meghe Institute of Higher Education & Research, Wardha, IND

**Keywords:** plyometric with pilates, sargent jump test, pilates training, plyometric training, basketball

## Abstract

Introduction

Basketball is an athletic court game sport played by five players from two sports teams each. Therefore, the objective of this study is aimed at gauging the potential impact of the combination of plyometrics and Pilates training along with the efficacy of these exercise regimes individually on raising vertical jump performance in basketball players.

Methodology

For this study, 45 subjects were enrolled and assigned into groups A, B, and C. Group A was given a plyometric program, group B was given a Pilates program, and group C was given a combination of plyometrics and Pilates program. All were tested for vertical jump and muscle endurance prior to starting the plyometric and Pilates training and plyometrics with Pilates program. All the subjects passed through six weeks of the training program and were retested. The program was given three days a week for six weeks.

Result

The data collected were statistically analyzed by applying the Wilcoxon signed-rank test and the one-way analysis of variance test. The analysis of variance in vertical jump ability among three groups was assessed. The average value of vertical jump ability before training was 260.60 for group A, 243.47 for group B, and 263 for group C. Training group C had a higher mean vertical jump value. All groups showed improvement in vertical jump, but group C (plyometrics with Pilates) showed greater improvement in the vertical jump height and trunk flexor and trunk extensor endurance test.

Conclusion

The finding suggested that group C (plyometrics with Pilates) is more effective than group A (plyometrics) and B (Pilates) in improving the vertical jump height.

## Introduction

Basketball is one of the most popular sports in which performance is improved by the combination of some specific skills and training. Therefore, it is crucial to adopt a superior training program as basketball is a complex sport that requires biomechanical and motor coordination competence to become proficient. For instance, sprinting, coordination, and jumping are of utmost significance for basketball players, and they depend upon power, pace, and endurance [1]. Basic basketball skills include fast direction changes, vertical leaps, and complexity of motor coordination both with and without a ball [2].

Enhancing vertical jump performance (VJP) is significant in many sports, and it is the outcome of muscle strength and speed [3]. The capability to produce explosive lower body power can be an important factor for attaining the maximum height in a jump. Certain training procedures are required to enhance the vertical jumping capability of players [4].

The vertical jump is a major constituent of numerous sports and games and can also predict performance in other sports in which it is not a major constituent. The impact of plyometrics on VJP has been studied broadly. In particular, some authors illustrated a notable rise in the vertical jump height after the plyometric procedure [5-9], whereas others reported futile [10]. 

Plyometric training is the most frequently used procedure among coaches and researchers to enhance vertical jump performance and leg strength in a plyometric exercise. The countermovement jump, drop, and squat jump are the utmost familiar plyometric training [11]. Plyometric training enhances muscular energy and strength, cooperation, and performance of players, in comparison to Pilates exercise depending on the postulate of trunk steadiness, which is known as core stability. The core is a box made up of the following muscles: the transverse abdominis anteriorly, the paraspinal (multifidus) posteriorly, the diaphragm above, and the pelvic floor below [12].

Pilates is a series of exercises aimed at building strength, flexibility, and muscular endurance. It re-improves the connection between mind and body. Pilates training empowers core muscles and enhances mobility, efficiency, and muscle power [13].

Pilates workouts can be categorized into two: mat and equipment workouts. The mat workouts were established by Joseph Pilates [13,14]. Mat workouts require members to sit or lie on their backs or stomach and use gravity to stabilize their core. Pilates enhances flexibility, builds muscle strength, and increases overall body control and endurance. It focuses on positioning, breathing, making a strong core, and enhancing coordination and equilibrium [14].

## Materials and methods

A three-arm parallel group randomized controlled trial was performed using simple random sampling with 1:1 recruitment that involved 45 male state-level basketball players who were already performing their routine training. The players were divided into three groups: group A was trained with plyometric exercises, group B was trained with Pilates exercises, and group C was trained with plyometric exercises along with Pilates exercises. Players in the age group of 18-35 years, who were state-level basketball male players with no traumatic or non-traumatic injury in the last one month and were willing to participate, were included in the study, whereas district-level players and players having recent traumatic or non-traumatic injuries were excluded from the study. Before commencing the intervention, approval from the Institutional Ethical Committee (IEC) was obtained with the IEC reference number SAIMS/22/1137 along with written informed consent from the players. This study was conducted at the National Basketball Academy, Indore, Madhya Pradesh. The duration of this study was six weeks. The training was provided three days a week (alternate days) in the morning, and the duration of the training was 45 minutes with a one-minute rest interval between each set. The assembled data were statistically inspected by analysis of variance (ANOVA). The outcome measure of the study was the Sargent jump, flexion, and extension endurance test of the trunk, which was tested before starting the training and after completion of training. The plyometric training protocol followed is shown in Table 1.

**Table 1:** Week-wise protocol of plyometric drills with training volume, repetitions, sets, and training intensity

Training Weeks	Training Volume	Plyometric Drills	Repetitions X Sets	Training intensity
Week- 1	85	Two feet ankle jump	15x2	Lower
		Onward skip	15x2	Lower
		Vertical leap of double leg	5x5	Lower
Week -2	110	Ankle jump of two feet	15x2	Lower
		Long jump by standing	6x5	Lower
		Sideways cone hops	15x2	Moderate
		Pair leg tuck jump	10x2	Moderate
Week -3	115	Two feet ankle hops	12x2	Lower
		Long jump by standing	6x4	Lower
		Sideways cone hops	12x2	Moderate
		Pair leg tuck jump	10x2	Moderate
		Double butt kick	8x3	Moderate
Week -4	105	Diagonal hops	8x4	Lower
		Double tuck jump	10x2	Moderate
		Lateral cone hops	10x2	Moderate
		Double leg butt kick	6x3	Moderate
		Single leg vertical jump	5x3	Higher
Week -5	110	Diagonal cone hops	7x3	Lower
		Standing long jump with lateral sprint	5x4	Moderate
		Lateral cone hops	5x2	Moderate
		Single leg bounding	5x2	Higher
		Front cone hops	10x2	Moderate
		Depth jumps	5x3	Higher
Week- 6	110	Diagonal cone hops	7x2	Lower
		Hexagon drill	12x2	Lower
		Double leg hops	8x3	Moderate
		Lateral cone hops	8x3	Moderate
		Depth jump	7x2	Higher

The Pilates training protocol started with lifting the leg upward (linear sitting). Afterward, balance sitting was achieved by shifting the legs unclosed. The next step was raising the legs and arm reversely (lying down aside), followed by relaxing heels and shoulder on a carton and another rump respectively. Another exercise included interchanging the opposite side with the elevated leg higher and arm (inclination and laying down). The next workout involved laying arms upward, which was succeeded by elevating the opposite leg backward and upward and arms reversely (lying down lift). Again, the elevation of the leg to one side (inclining and laying down) was continued by forward and upward movement of the upper leg (inclining and resting lower on the arm). The next step was performed as lifting the leg upward and backward (resting low on two arms on either side), accompanied by the elevation of the lower limbs above, backward and forward that was unclosed (on either side of two arms lying low). The last exercise was the elevation of the pelvis upward and forward followed by the raising leg upward and forward (squatting by lying down). 

The mean and standard deviation were calculated for the vertical jump ability of all three groups. The procedure involved warm up for 10 minutes, and then the athlete chalks the end of his fingertips and stands side onto the wall, keeping both feet remaining on the ground, and reaches up as high as possible with one hand and marks the wall with the tips of the fingers (M1). Then, from a static position, the athlete jumps as high as possible and marks the wall with the chalk on his fingers (M2). Following this, the assistant measured and recorded the distance between M1 and M2. The athlete repeated the test three times, and the assistant calculated the average of the recorded distances and used this value to assess the athlete's performance. The obtained data were inspected by utilizing the one-way ANOVA test.

## Results

Table 2 expressed the analysis of variance in vertical jump ability among three groups. The average value of vertical jump ability before training was 260.60 for group A, 243.47 for group B, and 263 for group C. After administration of training, it was 268.27 for group A, 247.07 for group B, and 273.27 for group C. Group C had a higher mean vertical jump value. All groups showed improvement in the vertical jump, but group C (plyometrics with Pilates) showed greater improvement in the vertical jump height and trunk flexor and trunk extensor endurance test.

**Table 2:** Comparison between three groups (Plyometrics- Group A, Pilates- Group B, and Plyometrics with Pilates- Group C) in different applied tests *Kruskal-Wallis one-way ANOVA test for comparison of more than two groups SD: Standard deviation; ANOVA: analysis of variance

Variable	Plyometric (Mean ± SD)- Group A	Pilates (Mean ± SD)- Group B	Plyometric with Pilates (Mean ± SD)- Group C	p-value*
Age	16.53 ± 1.598	16.87 ± 1.125	16.93 ± 1.981	0.719
Pre-Sargent jump test (M1)	224.33 ± 8.269	206.80 ± 16.502	225 ± 8.920	0.001
Post-Sargent jump test (M1)	224.33 ± 8.269	206.80 ± 16.502	225 ± 8.920	0.001
Pre-Sargent jump test (M2)	260.60 ± 9.470	243.47 ± 19.530	263 ± 11.856	0.007
Post-Sargent jump test (M2)	268.27 ± 9.610	247.07 ± 19.495	273.27 ± 12.262	0.001
Pre-Sargent jump test (M2-M1)	36.27 ± 8.689	36.67 ± 17.442	38 ± 9.509	0.542
Post-Sargent jump test (M2-M1)	43.93 ± 8.746	40.27 ± 17.421	48.27 ± 9.750	0.027
Pre-trunk flexor endurance test	22.67 ± 15.559	25.73 ± 7.005	20.40 ± 4.867	0.034
Post-trunk flexor endurance test	74.47 ± 15.679	56.07 ± 7.235	95.40 ± 4.867	0.001
Pre-trunk extensor endurance test	30.87 ± 13.820	31.27 ± 8.379	31.07 ± 12.297	0.898
Post-trunk extensor endurance test	81.47 ± 13.923	61.67 ± 8.641	106.47 ± 12.340	0.001

## Discussion

The current study found that vertical jumping ability is increased via plyometrics with pilates training. Other researchers have also done research on plyometric training and pilates training; both were effective in the vertical jumping ability of players according to Brown et al. [15], Singh et al. [4], Silva et al. [16], and El Sayed et al. [14], but plyometrics showed better improvement in vertical jump according to Parekh et al. [17]. The current study showed that when plyometrics is given in combination with Pilates, it showed more improvement in vertical jump compared to them individually. Because of the actin and myosin’s contractile elements that are fastening sarcomere, they act as crucial functions in motor command and force development during plyometrics [15,17].

Enhancing VJP is essential in many sports, and as a result, VJP is associated with success in many sports [3]. It is the result of muscular strength and speed. The ability to generate explosive lower body power may play a significant role in determining the jumper's greatest height. In order to improve players' capacity to jump vertically, certain training techniques are necessary [4]. The vertical jump is a major constituent of numerous sports and games and can also predict performance in other sports in which it is not a major constituent. The impact of plyometrics on VJP has been studied broadly. In particular, some authors illustrated a notable rise in the vertical jump height after the plyometric procedure [5-9], whereas others reported futile [10].

Plyometrics comprises the capability of fibers of the muscles to produce greater force and the resulting force generation by pre-stretching the physiological length-tension curve of the muscle-tendon unit [18]. Another rationale for enhancing the vertical jump by body proprioceptors involves stretch receptors, neurotendinous spindle, and mechano-receptors present in joint ligaments and capsules, which are expected to activate the receptors and can lead to agonist and antagonistic muscle facilitation, cessation, and alterations. These mechanisms have been proposed to enhance the vertical jump using Pilates exercises for biological efficiency and kinetic chain activity. Players voluntarily hold their breath during any athletic activity. This can affect performance by reducing air intake, oxygen uptake, and energy.

Controlling core robustness, equilibrium, and mobility will enhance all active chains of upper and lower limb activity. Pilates exercises focus on the core musculature and are a series of exercises aimed at building strength, flexibility, and muscular endurance. It re-improves the connection between mind and body. Pilates training empowers core muscles and enhances mobility, efficiency, and muscle power [13]. Pilates workout increases the power and forbearance of core muscles, and it works as a bridge between the upper and lower body. It focuses on positioning, breathing, making a strong core, and enhancing coordination and equilibrium [14]. Due to the increased power and forbearance strength of core muscles, Pilates enhances the shift of the lower body forces to the upper body respectively [19]. When plyometric training is combined with Pilates training, it results in greater vertical jump improvement due to improved biological efficiency, kinetic chain activation, and neuromuscular activation; a higher stretch-shortening cycle; and increased strength of lower limb muscles and core muscles, all of which lead to improved vertical jump ability [20].

## Conclusions

This study concludes that the VJP of the male basketball players belonging to group C (plyometrics with Pilates) exhibited greater improvements after uncovering significant differences in before and after tests of jumping ability. Furthermore, future studies can be carried out by comparing the mean variables from their study with already reported ones. To summarize the present study, the efficacious training method was found to be plyometrics along with Pilates to enhance VJP. This research was organized on a small magnitude with fewer sample size, mental and surrounding facets were not considered, and solely male players were enrolled in this study.
